# Inverse associations between light-to-moderate alcohol intake and lipid-related indices in patients with diabetes

**DOI:** 10.1186/1475-2840-12-104

**Published:** 2013-07-17

**Authors:** Tomoko Shimomura, Ichiro Wakabayashi

**Affiliations:** 1 Department of Environmental and Preventive Medicine, Hyogo College of Medicine, Mukogawa-cho 1-1, Nishinomiya, Hyogo 663-8501, Japan

## Abstract

**Background:**

Dyslipidemia is a common complication in patients with diabetes and is involved in being prone to cardiovascular disease. The risk of coronary artery disease is known to be lower in light-to-moderate drinkers than in abstainers. The aim of this study was to clarify whether and how alcohol drinking influences the lipid-related indices, good predictors for cardiovascular disease, such as the ratio of LDL cholesterol to HDL cholesterol (LDL-C/HDL-C ratio), the ratio of triglycerides to HDL cholesterol (TG/HDL-C ratio), and the lipid accumulation product (LAP), in patients with diabetes.

**Methods:**

The subjects were men with diabetes (n = 1477; mean age, 54.0 years) and they were divided into non-, light (< 22 g ethanol/day), moderate (≥ 22 and < 44 g ethanol/day) and heavy (≥ 44 g ethanol/day) drinkers. The relationships between alcohol intake and the lipid-related indices were investigated by the multivariate analyses with adjustment for age, smoking, regular exercise and drug therapy for diabetes.

**Results:**

The odds ratio (OR) vs. nondrinkers for high LDL-C/HDL-C ratio tended to be lower with an increase in alcohol intake (OR with 95% confidence interval (CI): 0.80 [0.50-1.29] in light drinkers; 0.24 [0.15-0.38] in moderate drinkers and 0.10 [0.05-0.19] in heavy drinkers). Alcohol intake showed an inverse association with a high TG/HDL-C ratio (OR with 95% CI vs. nondrinkers for high TG/HDL-C ratio: 0.54 [0.36-0.80] in light drinkers; 0.73 [0.56-0.97] in moderate drinkers and 0.72 [0.53-0.98] in heavy drinkers) and a J-shaped relationship with a high LAP (OR with 95% CI vs. nondrinkers for high LAP: 0.66 [0.43-1.02] in light drinkers; 0.82 [0.61-1.10] in moderate drinkers, and 1.29 [0.95-1.77] in heavy drinkers). Similar associations between alcohol intake and the lipid indices were obtained in a covariance analysis.

**Conclusions:**

In patients with diabetes, light-to-moderate alcohol consumption is inversely associated with lipid-related indices, and this correlates with previous findings of cardiovascular risk reduction by modest drinking in patients with diabetes.

## Introduction

The risk of cardiovascular disease is known to be lower in light-to-moderate alcohol drinkers than in abstainers [1]. The effects of alcohol on lipid metabolism, especially the HDL cholesterol-elevating effects, are thought to greatly contribute to the cardioprotective action of alcohol [2]. On the other hand, excessive alcohol consumption has been shown to cause hypertriglyceridemia [3,4], which is a prevalent risk factor for cardiovascular disease [5-7]. With regard to mechanisms underlying the effects of alcohol on lipid metabolism, alcohol consumption has been shown to increase the activity of lipoprotein lipase and decrease the activity of cholesteryl ester transfer protein, resulting in elevation of HDL cholesterol [8]. The hypertriglyceridemia induced by excessive alcohol drinking may be mainly due to an increase in the synthesis of large VLDL particles in the liver [4].

Diabetes mellitus is a major risk factor for cardiovascular disease. An inverse association between moderate drinking and the incidence of diabetes has been suggested in recent systemic reviews [9,10], although it is still under debate whether there is a causal relationship between alcohol and diabetes. Patients with diabetes are prone to have dyslipidemia such as high triglyceride levels, low HDL cholesterol levels, and a preponderance of small dense LDL particles, and a high prevalence of dyslipidemia contributes to the pathogenesis of atherosclerotic macrovascular disease in patients with diabetes [11,12].

Several lipid-related indices have been proposed by previous studies to monitor patients for prediction of cardiovascular disease. In addition to the ratio of LDL cholesterol to HDL cholesterol (LDL-C/HDL-C ratio), a classical atherogenic index [13], the ratio of triglycerides to HDL cholesterol (TG/HDL-C ratio) has been shown to be a good discriminator for cardiovascular risk [14,15] and to reflect atherogenic small dense LDL particles [16]. The TG/HDL-C ratio has also been demonstrated to be associated with insulin resistance [17] and metabolic syndrome [18]. Lipid accumulation product (LAP), a continuous marker of lipid over-accumulation calculated by using the waist circumference and triglyceride levels, has recently been proposed to be a good predictor for cardiovascular disease [19] and diabetes [20]. Although light-to-moderate drinking has been reported to show inverse associations with these lipid-related indices in a general population [21-23], it is unknown whether and how alcohol drinking influences the lipid indices in patients with diabetes. The purpose of this study was therefore to clarify the relationships between alcohol intake and lipid indices, including LDL-C/HDL-C ratio, TG/HDL-C ratio and LAP, in patients with diabetes.

## Methods

### Subjects

A cross-sectional study was performed using a local population-based database. The subjects in the original database of the health checkup were male workers aged from 35 to 70 years (n = 37693) who had received periodic health examinations at their workplaces in Yamagata Prefecture in Japan. Subjects with diabetes (n = 1477) were extracted from the database according to the definition of diabetes given below. Subjects with diabetes were defined as those showing high hemoglobin A_1C_ levels (≥ 6.5%), according to the recent criteria for diagnosis of diabetes by the American Diabetes Association [24], and/or having a current history of drug therapy for diabetes. Those receiving treatment for dyslipidemia were excluded from the subjects. All of the subjects were of Japanese origin. Subjects who were receiving treatment for any illness were requested to state the names of the diseases in a questionnaire at the health checkup. This study was approved by the Ethics Committee of Yamagata University School of Medicine. The histories of alcohol consumption, cigarette smoking and regular exercise were also surveyed by questionnaires.

### Classification of drinker groups

The average alcohol consumption of each subject per week was reported on questionnaires during health examinations at each workplace. Since it is difficult to know the correct average alcohol consumption of occasional drinkers, only regular drinkers who drank almost every day were used as drinkers for the analysis in this study. The usual daily alcohol consumption was calculated in terms of the equivalent number of “go”, a traditional Japanese unit of sake (rice wine). The amounts of other alcoholic beverages, including beer, wine, whisky and shochu (a traditional Japanese distilled spirit), were converted and expressed as units of “go”. One go approximately corresponds to 180 ml of sake, 500 ml of beer, 240 ml of wine, 60 ml of whisky and 80 ml of shochu. The amount of alcohol consumed daily was categorized as “null”, “less than 1 go per day”, “1 go or more, but less than 2 go per day”, “2 go or more, but less than 3 go per day” and “3 go or more per day”. One “go” contains about 22 g of ethanol, and this amount was used to separate moderate drinkers from light drinkers since it is generally accepted that alcohol intake should be reduced to less than 20 – 30 g per day from the viewpoint of the prevention of hypertension [25,26]. The average daily alcohol intake (grams of ethanol per day) was then calculated. The subjects were divided into four groups according to the ethanol consumption per day (non-drinkers; light drinkers: < 22 g of ethanol per day; moderate drinkers: ≥ 22 g and < 44 g of ethanol per day; heavy drinkers: ≥ 44 g of ethanol per day).

### Measurements

The height and body weight were measured with the subjects wearing light clothes at the health checkup. The waist circumference was measured at the navel level according to the recommendation of the definition of the Japanese Committee for the Diagnostic Criteria of Metabolic Syndrome [27]. Fasted blood was sampled from each subject in the morning, and the serum triglycerides, HDL cholesterol and LDL cholesterol levels were measured by enzymatic methods using commercial kits: the pureauto S TG-N, cholestest N-HDL and cholestest LDL (Sekisui Medical Co., Ltd, Tokyo, Japan), respectively. The coefficients of variation for the reproducibility of each measurement were ≤ 3% for triglycerides, ≤ 5% for HDL cholesterol and ≤ 5% for LDL cholesterol. The cut-off values for a high LDL-C/HDL-C ratio and high TG/HDL-C ratio were defined as 3.5 and 3.75, respectively. The LAP was determined by using the triglycerides (TG) level and waist circumference (WC) as follows: LAP = TG (mmol/L) × (WC (cm) – 65) [19]. The values of LAP were arranged in ascending order, and the subjects were then divided into three groups of approximately equal sizes. Subjects in the highest tertile of LAP were defined as subjects with a high LAP, since a common cut-off value for LAP has not been confirmed. Hemoglobin A1c was measured by the NGSP (National Glycohemoglobin Standardization Program)-approved technique using the latex cohesion method with a commercial kit (Determiner HbA1c, Kyowa Medex, Tokyo, Japan). The coefficient of variation for reproducibility of the hemoglobin A1c measurement was ≤ 5%. Since the standards of hemoglobin A1c used for measurement are different in the NGSP method and JDS (the Japan Diabetes Society) method, the hemoglobin A1c values were calibrated by using a formula proposed by the JDS [28]: hemoglobin A1c (NGSP) (%) = 1.02 × hemoglobin A1c (JDS) (%) + 0.25%.

### Statistical analysis

The statistical analyses were performed using a computer software program (SPSS version 16.0 J for Windows, Chicago IL, USA). The percentages of smokers, subjects exercising regularly and subjects receiving drug therapy for diabetes were compared between each pair of groups using the chi-square test for independence. In the univariate analysis, means of each variable were compared among the groups by using an analysis of variance (ANOVA) followed by Scheffé’s F-test as a post-hoc test. In the multivariate analysis, the mean levels of each variable were compared by using an analysis of covariance (ANCOVA), followed by Student’s *t*-test after Bonferroni correction. Since the triglycerides, TG/HDL-C ratio and LAP levels did not show a normal distribution, these parameters were compared between the groups non-parametrically by using the Kruskal-Wallis test, followed by the Steel-Dwass test as a post-hoc test in the univariate analysis (ANOVA), or were used after log-transformation in the multivariate analysis (ANCOVA). In the logistic regression analysis, the crude and adjusted odds ratios for a high LDL-C/HDL-C ratio, high TG/HDL-C ratio, high LAP or a combination of these high lipid indices were calculated. Age, smoking, regular exercise and drug therapy for diabetes were used as other explanatory variables or covariables in the multivariate analyses. In the analyses of variables other than waist circumference and LAP, body weight was also added to the explanatory variables and covariables. Probability (*p*) values less than 0.05 were defined as significant.

## Results

### Characteristics of the subject groups

Table 1 shows the results of comparison of the alcohol groups by a univariate analysis. Body weight and waist circumference were significantly lower and smaller, respectively, in light and moderate drinkers than in nondrinkers. The percentage of smokers was significantly higher in moderate and heavy drinkers than in nondrinkers, while there was no significant difference in the percentage of subjects doing regular exercise among the alcohol groups. The triglyceride level was significantly lower in light drinkers than in nondrinkers and was higher with a marginal significance (*p* = 0.053) in heavy drinkers than in nondrinkers. The HDL cholesterol level was significantly higher in light, moderate and heavy drinkers than in nondrinkers, while the LDL cholesterol level was significantly lower in moderate and heavy drinkers than in nondrinkers. The LDL-C/HDL-C ratio was significantly lower in light, moderate and heavy drinkers than in nondrinkers and tended to be lower with an increase in alcohol intake. The TG/HDL-C ratio was significantly lower in light and moderate drinkers than in nondrinkers. The LAP was significantly lower in light drinkers and significantly higher in heavy drinkers when compared with that in nondrinkers.

**Table 1:** **Characteristics of the nondrinkers**, **light drinkers**, **moderate drinkers and heavy drinkers in patients with diabetes**

	**Nondrinkers**	**Light drinkers**	**Moderate drinkers**	**Heavy drinkers**	**Overall**
Number	567	167	439	304	1477
Age (years)	52.4 ± 8.6	56.1 ± 6.4**	55.2 ± 7.1**	54.1 ± 6.6*	54.0 ± 7.7
Body weight (kg)	72.9 ± 15.6	69.4 ± 10.9*	68.6 ± 11.0**	71.0 ± 11.9	70.8 ± 13.2
Waist circumference (cm)	89.0 ± 11.6	86.9 ± 8.2	86.4 ± 8.5**	88.0 ± 8.8	87.8 ± 9.9
Smokers (%)	48.5	48.5	60.8**	59.2**	54.4
Regular exercise (%)	9.9	13.8	12.1	9.5	10.9
Drug therapy for diabetes (%)	52.9	54.5	50.3	44.4*	50.6
Hemoglobin A1c (%)	7.84 ± 1.72	7.68 ± 1.57	7.48 ± 1.41**	7.70 ± 1.44	7.69 ± 1.56
Triglycerides (mg/dl)	144 (93, 223)	120 (78, 175)**	131 (81, 216)	164 (99, 256)#	142 (90, 223)
HDL cholesterol (mg/dl)	47.1 ± 11.8	53.1 ± 13.5**	56.5 ± 14.7**	57.2 ± 14.7**	52.7 ± 14.3
LDL cholesterol (mg/dl)	124.9 ± 31.5	122.3 ± 32.2	115.7 ± 32.1**	111.1 ± 34.8**	119.0 ± 32.9
LDL-C/HDL-C ratio	2.81 ± 0.96	2.47 ± 0.93**	2.19 ± 0.82**	2.06 ± 0.82**	2.43 ± 0.94
TG/HDL-C ratio	3.16 (1.87, 5.58)	2.33 (1.42, 3.79)**	2.36 (1.31, 4.47)**	2.96 (1.65, 5.46)	2.77 (1.56, 5.00)
LAP	38.2 (18.7, 65.5)	28.2 (18.5, 50.4)**	32.6 (16.7, 58.5)	40.4 (22.0, 73.9)**	36.0 (18.5, 63.8)

The numbers of subjects in each group, percentage of subjects with a smoking habit, a habit of regular exercise or who received drug therapy for diabetes, the means with the standard deviations for each variable and the medians with 25th and 75th percentile values of each variable are shown. Symbols denote significant differences from nondrinkers (*, *p* < 0.05; **, *p* < 0.01). A marginally significant difference from nondrinkers (#, *p* = 0.053).

### Comparison of hemoglobin A1c among alcohol groups by the multivariate analysis

The hemoglobin A1c levels (means with standard errors) were 7.83 ± 0.06%, 7.78 ± 0.12%, 7.50 ± 0.07% and 7.64 ± 0.09% in nondrinkers, light drinkers, moderate drinkers and heavy drinkers, respectively. Hemoglobin A1c was significantly lower in moderate drinkers than in nondrinkers, and there was a tendency of an inverted J-shaped relationship between the alcohol intake and hemoglobin A1c level, although there were no significant differences in hemoglobin A1c between light drinkers and nondrinkers and between heavy drinkers and nondrinkers.

### Comparison of the variables determining the lipid-related indices among alcohol groups by the multivariate analysis

**Figure 1:**
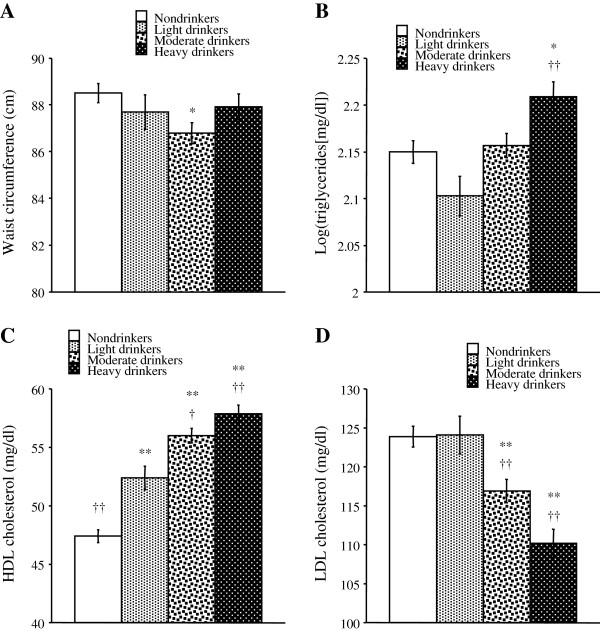
**Comparison of the variables determining the lipid indices among non-, light, moderate and heavy drinkers in patients with diabetes. **The mean levels of variables (**A**, waist circumference; **B**, log-transformed triglycerides; **C**, HDL cholesterol; **D**, LDL cholesterol) were calculated after adjustment for age, smoking, regular exercise and drug therapy for diabetes. Body weight was also added to covariates to calculate the means of the variables other than waist circumference. Symbols denote significant differences from nondrinkers (*, *p* < 0.05; **, *p* < 0.01) and light drinkers (†, *p* < 0.05; ††, *p* < 0.01).

Figure 1 shows the means of each variable determining the lipid indices calculated with adjustment for age, smoking, exercise and drug therapy for diabetes. Body weight was also adjusted to calculate the means of the variables other than waist circumference. Waist circumference was significantly smaller in moderate drinkers than in nondrinkers (Figure 1A). The log-transformed triglyceride levels were significantly higher in heavy drinkers than in nondrinkers and light drinkers (Figure 1B). The HDL cholesterol level was significantly higher in light, moderate and heavy drinkers than in nondrinkers and tended to be higher with an increase in alcohol intake (Figure 1C). The LDL cholesterol level was significantly lower in moderate and heavy drinkers than in nondrinkers and light drinkers (Figure 1D).

### Comparison of the lipid-related indices among alcohol groups by the multivariate analysis

**Figure 2:**
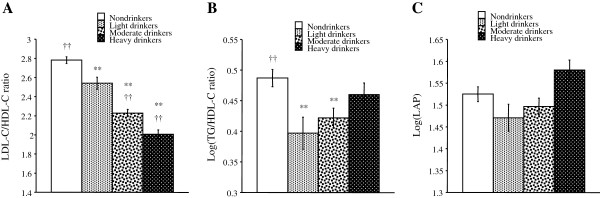
**Comparison of the lipid indices among non-, light, moderate and heavy drinkers in patients with diabetes.** The mean levels of variables (**A**, LDL-C/HDL-C ratio; **B**, log-transformed TG/HDL-C ratio; **C**, log-transformed LAP) were calculated after adjustment for age, smoking, regular exercise and drug therapy for diabetes. Body weight was also added to covariates to calculate the means of the variables other than LAP. Symbols denote significant differences from nondrinkers (**, *p* < 0.01) and light drinkers (†, *p* < 0.05; ††, *p* < 0.01).

The mean levels of each lipid index calculated with adjustment for the aforementioned confounders are shown in Figure 2. The LDL-C/HDL-C ratio was significantly lower in light, moderate and heavy drinkers than in nondrinkers and tended to be lower with an increase in alcohol intake (Figure 2A). The log-transformed TG/HDL-C ratio was significantly lower in light and moderate drinkers than in nondrinkers (Figure 2B). Log-transformed LAP was significantly higher in heavy drinkers than in light drinkers. Log-transformed LAP tended to be lower in light drinkers and higher in heavy drinkers compared with that in nondrinkers, although the differences were not significant (Figure 2C).

### Odds ratios for high lipid indices in each drinker group vs. the nondrinker group

Table 2 shows the odds ratios vs. nondrinkers for the high lipid indices. Both the crude and adjusted odds ratios for a high LDL-C/HDL-C ratio were significantly lower in moderate and heavy drinkers compared with a reference level of 1.00 and tended to be lower with an increase in alcohol intake. Both the crude and adjusted odds ratios for a high TG/HDL-C ratio were significantly lower in the light, moderate and heavy drinker groups compared with a reference level of 1.00, except for the crude odds ratio of heavy drinkers, which was marginally significant (*p* = 0.077). The crude odds ratios for a high LAP were significantly lower in light and moderate drinkers compared with a reference level of 1.00, and the corresponding adjusted odds ratio also tended to be lower than the reference level. On the other hand, the crude and adjusted odds ratios for a high LAP of heavy drinkers vs. nondrinkers tended to be higher than the reference level, although the differences were not significant.

**Table 2:** **The odds ratios for the high LDL**-**C**/**HDL**-**C ratio**, **high TG**/**HDL**-**C ratio and high LAP of the light**, **moderate and heavy drinker groups vs**. **the nondrinker group in subjects with diabetes**

	**Nondrinkers**	**Light drinkers**	**Moderate drinkers**	**Heavy drinkers**
High LDL-C/HDL-C ratio				
Crude OR	1.00	0.68 (0.43-1.07)	0.22 (0.14-0.34)**	0.11 (0.05-0.21)**
Adjusted OR	1.00	0.80 (0.50-1.29)	0.24 (0.15-0.38)**	0.10 (0.05-0.19)**
High TG/HDL-C ratio				
Crude OR	1.00	0.47 (0.32-0.69)**	0.65 (0.50-0.84)**	0.77 (0.58-1.03)##
Adjusted OR	1.00	0.54 (0.36-0.80)**	0.73 (0.56-0.97)*	0.72 (0.53-0.98)*
High LAP				
Crude OR	1.00	0.51 (0.34-0.76)**	0.73 (0.56-0.96)*	1.23 (0.92-1.63)
Adjusted OR	1.00	0.66 (0.43-1.02)#	0.82 (0.61-1.10)	1.29 (0.95-1.77)

The crude and adjusted odds ratios (ORs) are shown with their 95% confidence intervals indicated in the parentheses. The adjusted odds ratios for the high LDL-C/HDL-C ratio, high TG/HDL-C ratio, and high LAP of each drinker group vs. nondrinkers were calculated using age, smoking, regular exercise and drug therapy for diabetes as other explanatory variables. Body weight was also used as an explanatory variable to calculate the adjusted odds ratios for high LDL-C/HDL-C ratio and high TG/HDL-C ratio. Symbols denote significantly lower odds ratios compared with a reference level of 1.00 (*, *p* < 0.05; **, *p* < 0.01). Marginally significant differences from the reference level (#, *p* = 0.060; ##, *p* = 0.077).

### Odds ratios for accumulation of high lipid indices in each drinker group vs. the nondrinker group

As shown in Table 3, both the crude and adjusted odds ratios of light and moderate drinkers vs. nondrinkers for ≥ one high lipid index, ≥ two high lipid indices or three high lipid indices were significantly lower than a reference level of 1.00, except for a marginal significance of the adjusted odds ratio of light drinkers vs. nondrinkers for three high lipid indices (*p* = 0.091). The crude and adjusted odds ratios of heavy drinkers vs. nondrinkers for three high lipid indices were also significantly lower than the reference level (Table 3), and these very low odds ratios may reflect the very low odds ratios for the high LDL-C/HDL-C ratio of heavy drinkers vs. nondrinkers (Table 2).

**Table 3:** **The odds ratios for the accumulation of high lipid**-**related indices** (**high LDL**-**C**/**HDL**-**C ratio**, **high TG**/**HDL**-**C ratio and high LAP**) **of the light**, **moderate and heavy drinker groups vs**. **the nondrinker group of subjects with diabetes**

	**Nondrinkers**	**Light drinkers**	**Moderate drinkers**	**Heavy drinkers**
≥ 1 high index				
Crude OR	1.00	0.54 (0.38-0.77)**	0.57 (0.44-0.73)**	0.82 (0.62-1.08)
Adjusted OR	1.00	0.63 (0.43-0.91)*	0.60 (0.46-0.79)**	0.79 (0.59-1.06)
≥ 2 high indices				
Crude OR	1.00	0.51 (0.34-0.76)**	0.59 (0.45-0.78)**	0.80 (0.60-1.08)
Adjusted OR	1.00	0.63 (0.42-0.96)*	0.63 (0.47-0.85)**	0.76 (0.55-1.05)
3 high indices				
Crude OR	1.00	0.36 (0.17-0.76)**	0.30 (0.18-0.52)**	0.12 (0.05-0.30)**
Adjusted OR	1.00	0.51 (0.24-1.11)#	0.35 (0.20-0.61)**	0.11 (0.04-0.27)**

The crude and adjusted odds ratios (ORs) are shown with their 95% confidence intervals indicated in the parentheses. The odds ratios vs. nondrinkers for ≥ 1, ≥ 2 or 3 high indices from the high LDL-C/HDL-C ratio, high TG/HDL-C ratio and high LAP in each drinker group were calculated. In the multivariate analysis, age, smoking, regular exercise and drug therapy for diabetes were used as other explanatory variables. Symbols denote significantly lower odds ratios compared with a reference level of 1.00 (*, *p* < 0.05; **, *p* < 0.01). A marginally significant difference from the reference level (#, *p* = 0.091).

## Discussion

The results of the covariance analysis and logistic regression analysis indicated that there is a linear inverse relationship between alcohol intake and the LDL-C/HDL-C ratio, a nonlinear inverse relationship between alcohol intake and the TG/HDL-C ratio, and a J-shaped relationship between alcohol intake and LAP. The findings of favorable associations between light-to-moderate drinking and lipid indices were reflected by the results showing an inverse association between alcohol intake and an accumulated risk of a high lipid index (Table 3). Therefore, in patients with diabetes, light-to-moderate alcohol consumption should lower these lipid-related indices, while the relationships between heavy alcohol intake and lipid indices differ by each lipid index. These associations between alcohol intake and lipid indices were similar to those found in a general population of Japanese subjects in previous studies [21-23].

This is the first study in which the relationships of alcohol intake with different lipid indices in patients with diabetes were compared. Although the TG/HDL-C ratio was higher in heavy drinkers than in light drinkers in the present study, a recent study by a Belgian group using 585 male patients with type 2 diabetes showed a linear inverse relationship between alcohol intake and the quintile of TG/HDL-C ratio in a univariate analysis [29]. A possible explanation for this discrepancy in the relationship between the TG/HDL-C ratio and heavy drinking is an ethnicity-dependent difference in the relationship between alcohol and blood lipids.

The lipid indices used in this study were determined by two variables out of triglycerides, HDL cholesterol, LDL cholesterol and waist circumference. A decrease in the LDL-C/HDL-C ratio with an increase in alcohol intake is reasonable, since HDL cholesterol and LDL cholesterol increased and decreased, respectively, with an increase in alcohol intake. While there was a positive linear association between alcohol intake and HDL cholesterol, the triglyceride levels were slightly lower in light drinkers and higher in heavy drinkers when compared with those in nondrinkers. These relationships resulted in a lower TG/HDL-C ratio in light and moderate drinkers than in nondrinkers and a smaller difference in the TG/HDL-C ratio between heavy drinkers and nondrinkers compared with the differences between nondrinkers and light or moderate drinkers. LAP was significantly higher in heavy drinkers than in light drinkers and showed a J-shaped relationship with alcohol intake, although the difference in LAP between each drinker group and the nondrinker group was not statistically significant. The association between alcohol intake and LAP was due to the slightly smaller waist circumference and lower triglycerides in light drinkers than in nondrinkers and was due to higher triglycerides in heavy drinkers than in nondrinkers.

Although each lipid index examined in this study showed diverse relationships with heavy drinking, light-to-moderate alcohol intake was inversely associated with all of the lipid indices in patients with diabetes. This agrees with previous epidemiological findings that light-to-moderate alcohol consumption reduced the risk of coronary artery disease not only in a general population [1] but also in patients with diabetes [30,31]. However, needless to say, drinking alcohol even if lightly or moderately should not be recommended to patients with diabetes for the prevention of cardiovascular complications, since drinking could disturb diet therapy and induce hypoglycemia in patients receiving treatment with anti-diabetic drugs.

There are some limitations of this study. Diabetes was diagnosed by the level of hemoglobin A1c in the health check-up examination and a history of drug therapy for diabetes, as assessed in the questionnaire. Thus, there is a possibility of an informational bias regarding the diagnosis of diabetes. In addition, the type of diabetes was not identified for each subject of this study. However, the type for most of the subjects is expected to be type 2 diabetes because the prevalence of type 2 diabetes is speculated to be > 100-times higher than that of type 1 diabetes in middle-aged Japanese men according to statistics [32]. Although age, body weight, smoking and exercise were adjusted in the multivariate analyses, the relationships between alcohol intake and lipid indices may have possibly been confounded by other factors such as diet, nutrition and socioeconomic factors, which were not included in the database used in this study. In addition, there is a possibility of confounding by polymorphisms in alcohol-metabolizing enzymes such as ADH (alcohol dehydrogenase) and ALDH (aldehyde dehydrogenase). Polymorphisms of genes related to lipid metabolism have also been reported to interact with overweight/obesity to influence blood lipid levels [33]. Moreover, apolipoprotein E gene polymorphisms have been shown to be associated with the lipid profile and prevalence of type 2 diabetes [34]. Therefore, polymorphisms of not only alcohol-metabolizing enzyme genes, but also lipid-related genes, are possible confounders for the relationships between the alcohol intake and lipid indices in patients with diabetes. The subjects of this study were middle-aged Japanese men, and there is a possibility that the relationships between alcohol and the lipid indices are modified by age, gender and ethnicity. In fact, the TG/HDL-C ratio has been reported not to show a significant relation to insulin resistance in African-American women [35]. Finally, this study was cross-sectional in its design, and further prospective studies are needed to elucidate causality of the association between alcohol consumption and the lipid indices.

## Conclusion

In conclusion, in patients with diabetes, there were significant inverse associations between light-to-moderate alcohol consumption and lipid-related indices. This supports the hypothesis that mild alcohol consumption reduces the risk of cardiovascular disease through improving the lipid profile in patients with diabetes.

## Competing interests

The authors have indicated that they have no competing interest regarding the content of this article.

## Authors’ contributions

TS and IW contributed to the conception, design and planning of the study. IW was involved in the collection of the data. TS and IW were involved in the analysis and interpretation of the data, did the statistical analysis and wrote the manuscript. Both authors read and approved the final manuscript.

## References

[B1] Corrao G Rubbiati L Bagnardim V Zambon A Poikolainen K Alcohol and coronary heart disease: a meta-analysis Addiction 2000 95 1505 1523 10.1046/j.1360-0443.2000.951015056.x 11070527

[B2] Ellison RC Zhang Y Qureshi MM Knox S Arnett DK Province MA Investigators of the NHLBI Family Heart Study Lifestyle determinants of high-density lipoprotein cholesterol: the National Heart, Lung, and Blood Institute Family Heart Study Am Heart J 2004 147 529 535 10.1016/j.ahj.2003.10.033 14999205

[B3] Castelli WP Doyle JT Gordon T Hames CG Hjortland MC Hulley SB Kagan A Zukel WJ Alcohol and blood lipids. The cooperative lipoprotein phenotyping study Lancet 1977 2 8030 153 155 69778 10.1016/s0140-6736(77)90176-3

[B4] Van de Wiel A The effect of alcohol on postprandial and fasting triglycerides Int J Vasc Med 2012 2012 862504 10.1155/2012/862504 21961068 PMC3179875

[B5] Hokanson JE Austin MA Plasma triglyceride level is a risk factor for cardiovascular disease independent of high-density lipoprotein cholesterol level: a meta-analysis of population-based prospective studies J Cardiovasc Risk 1996 3 213 219 10.1097/00043798-199604000-00014 8836866

[B6] Durrington PN Triglycerides are more important in atherosclerosis than epidemiology has suggested Atherosclerosis 1998 141 Suppl 1 S57 S62 9888644 10.1016/s0021-9150(98)00219-6

[B7] Labreuche J Touboul PJ Amarenco P Plasma triglyceride levels and risk of stroke and carotid atherosclerosis: a systematic review of the epidemiological studies Atherosclerosis 2009 203 331 345 10.1016/j.atherosclerosis.2008.08.040 18954872

[B8] Hannuksela ML Rämet ME Nissinen AET Liisanantti MK Savolainen MJ Effects of ethanol on lipids and atherosclerosis Pathophysiology 2004 10 93 103 10.1016/j.pathophys.2003.10.009 15006415

[B9] Baliunas DO Taylor BJ Irving H Roerecke M Patra J Mohapatra S Rehm J Alcohol as a risk factor for type 2 diabetes: a systematic review and meta-analysis Diabetes Care 2009 32 2123 2132 10.2337/dc09-0227 19875607 PMC2768203

[B10] Howard AA Arnsten JH Gourevitch MN Effect of alcohol consumption on diabetes mellitus: a systematic review Ann Intern Med 2004 140 211 219 10.7326/0003-4819-140-6-200403160-00011 14757619

[B11] Mooradian AD Dyslipidemia in type 2 diabetes mellitus Nat Clin Pract Endocrinol Metab 2009 5 150 159 10.1038/ncpendmet1066 19229235

[B12] Dunn FL Management of dyslipidemia in people with type 2 diabetes mellitus Rev Endocr Metab Disord 2010 11 41 51 10.1007/s11154-010-9132-6 20221703

[B13] Kannel WB Lipids, diabetes, and coronary heart disease: insights from the Framingham Study Am Heart J 1985 110 1100 1107 10.1016/0002-8703(85)90224-8 4061265

[B14] Gaziano JM Hennekens CH O’Donnell CJ Breslow JL Buring JE Fasting triglycerides, high-density lipoprotein, and risk of myocardial infarction Circulation 1997 96 2520 2525 10.1161/01.CIR.96.8.2520 9355888

[B15] Jeppesen J Hein HO Suadicani P Gyntelberg F Relation of high TG-low HDL cholesterol and LDL cholesterol to the incidence of ischemic heart disease. An 8-year follow-up in the Copenhagen Male Study Arterioscler Thromb Vasc Biol 1997 17 1114 1120 10.1161/01.ATV.17.6.1114 9194762

[B16] Dobiášová M Frohlich J The plasma parameter log (TG/HDL-C) as an atherogenic index: correlation with lipoprotein particle size and esterification rate in apoB-lipoprotein-depleted plasma (FER_HDL_) Clin Biochem 2001 34 583 588 10.1016/S0009-9120(01)00263-6 11738396

[B17] McLaughlin T Reaven G Abbasi F Lamendola C Saad M Waters D Simon J Krauss RM Is there a simple way to identify insulin-resistant individuals at increased risk of cardiovascular disease? Am J Cardiol 2005 96 399 404 10.1016/j.amjcard.2005.03.085 16054467

[B18] Cordero A Laclaustra M León M Casasnovas JA Grima A Luengo E Ordoñez B Bergua C Bes M Pascual I Alegría E MESYAS Registry Investigators Comparison of serum lipid values in subjects with and without the metabolic syndrome Am J Cardiol 2008 102 424 428 10.1016/j.amjcard.2008.03.079 18678299

[B19] Kahn HS The “lipid accumulation product” performs better than the body mass index for recognizing cardiovascular risk: a population-based comparison BMC Cardiovasc Disord 2005 5 26 10.1186/1471-2261-5-26 16150143 PMC1236917

[B20] Kahn HS The lipid accumulation product is better than BMI for identifying diabetes: a population-based comparison Diabetes Care 2006 29 151 153 10.2337/diacare.29.01.06.dc05-1805 16373916

[B21] Wakabayashi I Increased body mass index modifies associations between alcohol intake and blood cholesterol profile Eur J Clin Invest 2012 42 179 185 10.1111/j.1365-2362.2011.02568.x 21770926

[B22] Wakabayashi I Inverse association between triglycerides-to-HDL-cholesterol ratio and alcohol drinking in middle-aged Japanese men J Stud Alcohol Drugs 2012 73 998 1004 23036219 10.15288/jsad.2012.73.998

[B23] Wakabayashi I Relationship between alcohol intake and lipid accumulation product in middle-aged men Alcohol Alcohol*(in press)* 10.1093/alcalc/agt032 23592501

[B24] Anonymous; American Diabetes Association Diagnosis and classification of diabetes mellitus Diabetes Care 2010 33 Suppl 1 S62 S69 20042775 10.2337/dc10-S062 PMC2797383

[B25] Mancia G De Backer G Dominiczak A Cifkova R Fagard R Germano G Grassi G Heagerty AM Kjeldsen SE Laurent S Narkiewicz K Ruilope L Rynkiewicz A Schmieder RE Boudier HA Zanchetti A Vahanian A Camm J De Caterina R Dean V Dickstein K Filippatos G Funck-Brentano C Hellemans I Kristensen SD McGregor K Sechtem U Silber S Tendera M Widimsky P Management of Arterial Hypertension of the European Society of Hypertension; European Society of Cardiology 2007 Guidelines for the management of arterial hypertension: the task force for the management of arterial hypertension of the European Society of Hypertension (ESH) and of the European Society of Cardiology (ESC) J Hypertens 2007 25 1105 1187 10.1097/HJH.0b013e3281fc975a 17563527

[B26] Ogihara T Kikuchi K Matsuoka H Fujita T Higaki J Horiuchi M Imai Y Imaizumi T Ito S Iwao H Kario K Kawano Y Kim-Mitsuyama S Kimura G Matsubara H Matsuura H Naruse M Saito I Shimada K Shimamoto K Suzuki H Takishita S Tanahashi N Tsuchihashi T Uchiyama M Ueda S Ueshima H Umemura S Ishimitsu T Rakugi H Japanese Society of Hypertension Committee The Japanese Society of Hypertension Guidelines for the Management of Hypertension (JSH 2009) Hypertens Res 2009 32 3 107 19300436

[B27] Anonymous Metabolic syndrome-definition and diagnostic criteria in Japan J Jpn Soc Int Med 2005 94 794 809 in Japanese 10.2169/naika.94.794

[B28] Kashiwagi A Kasuga M Araki E Oka Y Hanafusa T Ito H Tominaga M Oikawa S Noda M Kawamura T Sanke T Namba M Hashiramoto M Sasahara T Nishio Y Kuwa K Ueki K Takei I Umemoto M Murakami M Yamakado M Yatomi Y Ohashi H Committee on the Standardization of Diabetes Mellitus-Related Laboratory Testing of Japan Diabetes Society (JDS) International clinical harmonization of glycated hemoglobin in Japan: from Japan diabetes society to national glycohemoglobin standardization program values Diabetol Int 2012 3 8 10 10.1007/s13340-012-0069-8 PMC4014931 24843544

[B29] Hermans MP Ahn SA Rousseau MF log(TG)/HDL-C is related to both residual cardiometabolic risk and β-cell function loss in type 2 diabetes males Cardiovasc Diabetol 2010 9 88 10.1186/1475-2840-9-88 21156040 PMC3020173

[B30] Anonymous; International Diabetes Federation Diabetes Atlas 2009 4 Available at: http://www.diabetesatlas.org 35914061

[B31] Valmadrid CT Klein R Moss SE Klein BEK Cruickshanks KJ Alcohol intake and the risk of coronary heart disease mortality in persons with older-onset diabetes mellitus JAMA 1999 282 239 246 10.1001/jama.282.3.239 10422992

[B32] Ajani UA Gaziano JM Lotufo PA Liu S Hennekens CH Buring JE Manson JE Alcohol consumption and risk of coronary heart disease by diabetes status Circulation 2000 102 500 505 10.1161/01.CIR.102.5.500 10920060

[B33] Yin RX Wu DF Miao L Aung LHH Cao XL Yan TT Long XJ Liu WY Zhang L Li M Several genetic polymorphisms interact with overweight/obesity to influence serum lipid levels Cardiovasc Diabetol 2012 11 123 10.1186/1475-2840-11-123 23039238 PMC3508802

[B34] Chaudhary R Likidlilid A Peerapatdit T Tresukosol D Srisuma S Ratanamaneechat S Sriratanasathavorn C Apolipoprotein E gene polymorphism: effects on plasma lipids and risk of type 2 diabetes and coronary artery disease Cardiovasc Diabetol 2012 11 36 10.1186/1475-2840-11-36 22520940 PMC3372424

[B35] Sumner AE Harman JL Buxbaum SG Miller BV 3rd Tambay AV Wyatt SB Taylor HA Rotimi CN Sarpong DF The triglyceride/high-density lipoprotein cholesterol ratio fails to predict insulin resistance in African-American women: an analysis of Jackson Heart Study Metab Syndr Relat Disord 2010 8 511 514 10.1089/met.2010.0028 20715971 PMC3125564

